# A preliminary study on ^226^Ra, ^232^Th, ^40^K and ^137^Cs activity concentrations in vegetables and fruits frequently consumed by inhabitants of Elazığ Region, Turkey

**DOI:** 10.1007/s10967-012-1995-4

**Published:** 2012-07-25

**Authors:** Cumhur Canbazoğlu, Mahmut Doğru

**Affiliations:** 1 Department of Physics, Faculty of Arts and Sciences, Kilis 7 Aralık University, 79000 Kilis, Turkey; 2 Department of Physics, Faculty of Arts and Sciences, Bitlis Eren University, Bitlis, Turkey

**Keywords:** Effective dose, Food stuff, Internal dose, Elazığ

## Abstract

Determining radioactivity levels in foodstuffs is of great importance for the protection of human health. In addition, the literature includes few studies related to this subject in Turkey. In this study, gamma spectroscopic system was used in order to measure ^226^Ra, ^232^Th, ^40^K and ^137^Cs activity concentrations in vegetables and fruits produced in Elazığ Region. The average activity concentrations in vegetables was calculated as 0.64 ± 0.26 Bq kg^−1^ for ^226^Ra, 0.65 ± 0.14 Bq kg^−1^ for ^232^Th, 13.98 ± 1.22 Bq kg^−1^ for ^40^K, and 0.54 ± 0.04 Bq kg^−1^ for ^137^Cs. The average activity concentrations in fruits were 1.52 ± 0.34, 0.98 ± 0.23, 18.66 ± 1.13 and 0.59 ± 0.16 Bq kg^−1^, respectively for ^226^Ra, ^232^Th, ^40^K and ^137^Cs. Total committed effective dose value was determined as 20 and 30.55 μSv y^−1^, respectively for vegetables and fruits. The findings were compared with previous data reported for Turkey and other regions of the world.

## Introduction

Natural radionuclide concentrations in environmental samples varies according to geographical and geological factors [1]. Natural sources of radioactivity in the environment are called naturally occurring radioactive materials, and are categorized as being of terrestrial or cosmic origin [2]. Humans are exposed to both internal and external radiation from these natural sources. Internal exposure occurs through the intake of terrestrial radionuclides through inhalation or ingestion. Inhalation exposure dose results from the existence of dust particles in air, including radionuclides from ^238^U and ^232^Th decay series. The biggest contribution to inhalation exposure comes from short half-life decay products of radon. Ingestion exposure dose mostly results from ^238^U and ^232^Th series radionuclides and ^40^K in drinking water and foodstuff. In addition, ^137^Cs is the most important fission product released to the environment as a result of nuclear activities, because this radionuclide rapidly passes to foodstuffs and creates a dose effect [3]. The literature includes this type of studies [4–10]. The aim of this study is to determine the exposure dose of ^226^Ra, ^232^Th, ^40^K and ^137^Cs radionuclide concentrations in fruits and vegetables produced in the Elazığ Region of Turkey, which are frequently consumed by local residents. The significance of the study is that it is the first study to determine the background radiation levels in such food products in this region and will provide data for future studies and in case of a nuclear accident (as in Chernobyl nuclear accident) or nuclear fallout, to determine level of contamination.

**Fig. 1:**
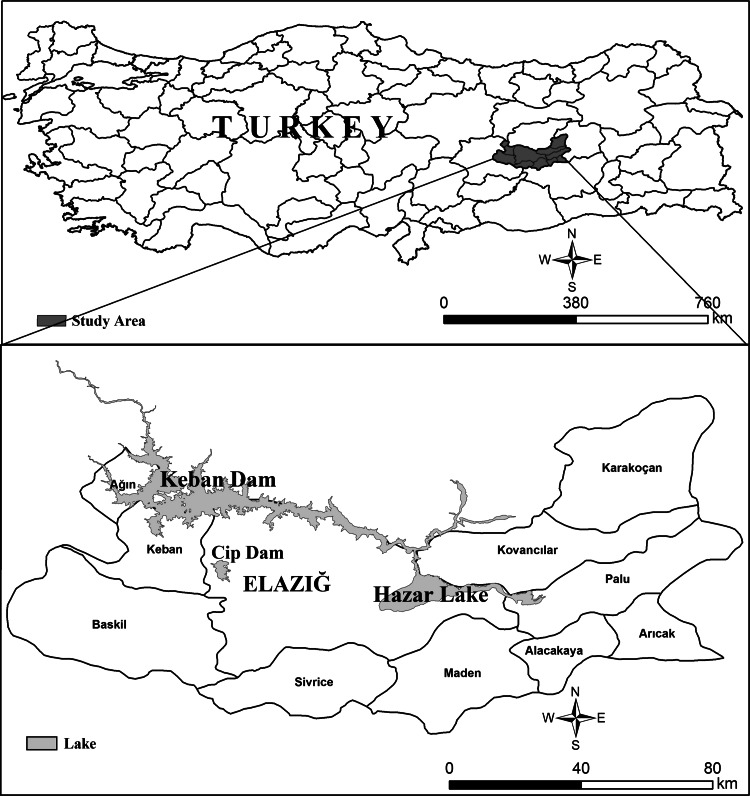
Map of Turkey showing the study area

The province of Elazığ is located in the Eastern Anatolian Region, between longitude 38°30′–40°21′E and latitude 38°17′–39°11′N. Its surface area is 9,151 km^2^ and the average altitude is 1,067 m. The region is divided into 11 administrative regions, with a total population of 540,000 (Fig. 1). Approximately 50 % of the province consists of grasslands, 28 % is agricultural land, 12 % forest, and 10 % is dams and lakes. A continental climate prevails; winters are cold and snowy, and summers are hot and arid. The province is rich in mineral resources, and mining activities include copper, fluoride, chalcopyrite, zinc, lead, chrome, manganese, molybdenum, iron and wolfram [11].

## Materials and methods

### Radioactivity measurements in vegetable and fruit samples

Samples of fruits and vegetables produced and frequently consumed in the region were provided from a public market. Any soil or foreign materials on the samples were removed so that they were suitable for consumption, divided into small pieces, and washed under distilled water. They were kept at room temperature for 3 months without allowing any contamination and then totally oven-dried at 105 °C. Afterwards, incineration was applied, which was realized through dry ashing. The temperature of the oven was increased to 250 °C and was continued until the samples were reduced to ash. The ashed samples were then homogenized and transferred into a plastic container (5 cm height × 5 cm diameter). Finally, the samples were sealed and stored for a period of about 1 month before counting, in order to allow equilibrium between ^226^Ra and its short-lived decay products.

The activity concentrations of ^226^Ra, ^232^Th, ^40^K and ^137^Cs radionuclides in vegetable and fruit samples were determined using a gamma spectroscopic system, comprising a 2″ × 2″ NaI(Tl) well-type detector and a detector surrounded by a cylindrical lead shield (thickness, diameter and length approximately 3.5, 13.7 and 15.5 cm, respectively). The detector window was made of aluminum of 0.50 mm thickness. Energy calibration of detector was performed by using ^60^Co (37 kBq) and ^226^Ra (370 kBq) point sources. Photopeak efficiency was 24 %. ^226^Ra, ^232^Th, ^40^K and ^137^Cs activity concentrations in vegetable and fruit were based on the detection of 609.3, 583, 1461 and 662 keV energy gamma rays transmitted by ^214^Bi, ^208^Tl, ^40^K and ^137^Cs, respectively.

### Calculation of activity concentration in vegetable and fruits

The activity concentrations in vegetable and fruit samples were calculated using Eq. (1)

1 $$A({\rm{Bq}}\,{\rm{kg}}^{{ - 1}} ) = \frac{C}{{M_{{\rm{s}}} \varepsilon P_{\gamma } }}$$

where* C* is the gamma ray count (number per second), ε is the detector efficiency of the specific gamma ray,* P*_γ_ is the absolute transition probability of gamma decay and* M*_s_ is the mass of the sample (kg) [12].

### Dose estimation

Ingestion dose occurring through the intake of radionuclides depends on the consumption rate of foodstuff and the concentration of the radionuclide involved. Ingestion dose is calculated with the Eq. (2) [3, 13, 14]

2 $$ H_{\text{T,r}} = \mathop \sum \nolimits \left( {U^{i} C_{\rm{r}}^{i} } \right)g_{\text{T,r}} $$

where* i* is foodstuff group, $U^{\it i}$ and $C_{\rm{r}}^{i}$ are annual consumption rate (kg) and radionuclide activity concentration (Bq kg^−1^), respectively for their coefficients, and $g_{\text{T,r}}$ is dose conversion coefficient for r radionuclide (Sv Bq^−1^). Dose conversion coefficients of ^226^Ra, ^232^Th, ^40^K and ^137^Cs radionuclides for the adult members of society are 4.5 × 10^−8^, 2.3 × 10^−7^, 6.2 × 10^−9^ and 1.3 × 10^−8^ Sv Bq^−1^, respectively [13, 15, 16].

## Results and discussion

Table 1 shows the natural and manmade radionuclide activity concentrations measured in samples of vegetables and fruits frequently consumed in Elazığ and its surrounding region. Minimum detectable activity values for vegetable and fruit samples were calculated as 0.02 Bq for ^232^Th and ^137^Cs; 0.03 Bq for ^226^Ra; and 0.1 Bq for ^40^K. Average activity concentrations of ^226^Ra, ^232^Th, ^40^K and ^137^Cs of vegetable samples were 0.64 Bq kg^−1^ (SD: 0.26), 0.65 Bq kg^−1^ (SD: 0.14), 13.98 Bq kg^−1^ (SD: 1.22) and 0.54 Bq kg^−1^ (SD: 0.04), respectively. The activity concentrations ranged between 0.11 and 0.99 Bq kg^−1^ for ^226^Ra; 0.47–0.84 Bq kg^−1^ for ^232^Th; 2.14–44.77 Bq kg^−1^ for ^40^K; and 0.17–0.79 Bq kg^−1^ for ^137^Cs. Average concentrations of ^226^Ra for fruits were 1.52 Bq kg^−1^ (SD: 0.34) and the values ranged between 0.73 and 2.81 Bq kg^−1^. ^232^Th concentrations ranged between 0.26 and 1.96 Bq kg^−1^ (average 0.98 Bq kg^1^, SD: 0.23). The average activities of ^40^K and ^137^Cs radionuclides were 18.66 Bq kg^−1^ (SD: 1.13) and 0.59 Bq kg^−1^ (SD: 0.16), respectively. ^40^K concentrations ranged between 1.34 and 35.49 Bq kg^−1^.

**Table 1:** Activity concentrations of vegetables and fruits

ID	Species	Scientific name	Activity concentrations of vegetables and fruits (Bq kg^−1^fresh weight)			
			^226^Ra	^232^Th	^40^K	^137^Cs
Vegetables						
F1	Bell pepper	*Capsicum annuum* L.	BDL	BDL	7.21 ± 0.91	0.48 ± 0.04
F2	Parsley	*Petroselinum crispum* (Mill.) Nyman & A.W. Hill	BDL	BDL	44.77 ± 1.90	BDL
F3	Scallion	*Allium cepa* L.	BDL	0.84 ± 0.17	29.41 ± 1.85	BDL
F4	Pumpkin	*Cucurbita moschata* Duchesne ex Poir.	BDL	BDL	2.14 ± 1.36	BDL
F5	Leek	*Allium ampeloprasum*	0.64 ± 0.37	BDL	10.02 ± 1.15	BDL
F6	Radish	*Raphanus sativus* L.	0.11 ± 0.04	0.47 ± 0.05	3.43 ± 0.34	0.17 ± 0.01
F7	Kale	*Brassica oleracea* Acephala	BDL	0.64 ± 0.24	5.78 ± 1.57	BDL
F8	Capsicum	*Capsicum annuum* L.	BDL	BDL	5.78 ± 0.63	BDL
F9	Cabbage	*Brassica oleracea* Capitata	0.95 ± 0.09	BDL	26.95 ± 0.95	BDL
F10	Tomato	*Solanum lycopersicum* L.	0.45 ± 0.08	0.64 ± 0.09	10.73 ± 0.70	BDL
F11	Eggplant	*Solanum melongena* L.	0.99 ± 0.19	BDL	16.57 ± 1.60	0.79 ± 0.06
F12	Lettuce	*Lactuca sativa* L.	BDL	BDL	30.93 ± 1.41	0.72 ± 0.05
F13	Spinach	*Spinacia oleracea* L.	0.80 ± 0.33	BDL	9.84 ± 0.92	BDL
F14	Peppermint	*Mentha spicata* L.	0.60 ± 0.36	BDL	2.22 ± 1.05	BDL
F15	Garden Cress	*Lepidium sativum* L.	0.54 ± 0.61	BDL	3.97 ± 1.90	BDL
Average			0.64 ± 0.26	0.65 ± 0.14	13.98 ± 1.22	0.54 ± 0.04
Fruits						
F16	Melon	*Cucumis melo* L.	1.01 ± 0.13	0.48 ± 0.13	35.49 ± 0.99	0.53 ± 0.04
F17	Pear	*Pyrus* spp.	BDL	1.96 ± 0.33	13.62 ± 1.60	BDL
F18	Quince	*Cydonia oblonga* Mill.	2.81 ± 0.45	1.14 ± 0.31	23.01 ± 1.40	0.64 ± 0.27
F19	Grapes	*Vitis vinifera* L.	BDL	0.26 ± 0.12	1.34 ± 0.63	BDL
F20	Watermelon	*Citrullus lanatus* (Thunb.) Matsum & Nakai	BDL	BDL	34.44 ± 0.88	BDL
F21	Apple	*Malus domestica* Borkh.	0.73 ± 0.45	1.04 ± 0.26	4.04 ± 1.25	BDL
Average			1.52 ± 0.34	0.98 ± 0.23	18.66 ± 1.13	0.59 ± 0.16

*BDL* below detection limit

Effective dose values exposed due to radionuclides taken into body through the consumption of fruit and vegetable samples are shown in Table 2. Primarily, average activity concentration (Bq kg^−1^) for each radionuclide was multiplied by food consumption rate, and annual activity intake value was determined in Bq unit. Food consumption rate was taken as 73 kg a^−1^ for both fruits and vegetables. This value represents the average consumption for Turkey [17]. The effective dose value was then determined by multiplying annual activity intake value by effective dose coefficient. Effective dose values of fruit samples for all radionuclides (^226^Ra, ^232^Th, ^40^K and ^137^Cs) were higher than those for vegetable samples. Average effective exposure dose through the consumption of vegetable samples were 2.12 μSv y^−1^ (SD: 0.86), 11.04 μSv y^−1^ (SD: 2.3), 6.33 μSv y^−1^ (SD: 0.55) and 0.51 μSv y^−1^ (SD: 0.04), respectively for ^226^Ra, ^232^Th, ^40^K and ^137^Cs. Effective dose values of ^226^Ra, ^232^Th, ^40^K and ^137^Cs ranged between 0.36 and 3.25, 7.89 and 14.10, 0.97 and 20.26 and 0.16 and 0.75 μSv y^−1^, respectively. Average effective doses through the consumption of fruit samples were 4.99 μSv y^−1^ (SD: 1.13), 16.56 μSv y^−1^ (SD: 3.91), 8.44 μSv y^−1^ (SD: 0.52) and 0.56 μSv y^−1^ (SD: 0.16), respectively for ^226^Ra, ^232^Th, ^40^K and ^137^Cs. Dose values ranged between 2.40 and 9.23 μSv y^−1^ for ^226^Ra; 4.37 and 32.91 μSv y^−1^ for ^232^Th; and 0.61–16.06 μSv y^−1^ for ^40^K.

**Table 2:** Dose coefficients and committed effective dose values for ^226^Ra, ^232^Th, ^40^K and ^137^Cs

Radioisotopes	Activity intake (Bq)	Effective dose coefficient (μSv Bq^−1^)	Committed effective dose (μSv y^−1^)	
			Range	Average
Vegetables				
^226^Ra	47 ± 19	0.045	0.36 ± 0.13–3.25 ± 0.62	2.12 ± 0.86
^232^Th	48 ± 10	0.23	7.89 ± 0.84–14.10 ± 2.85	11.04 ± 2.3
^40^K	1021 ± 89	6.2 × 10^−3^	0.97 ± 0.62–20.26 ± 0.86	6.33 ± 0.55
^137^Cs	39 ± 3	1.3 × 10^−2^	0.16 ± 0.01–0.75 ± 0.06	0.51 ± 0.04
Fruits				
^226^Ra	111 ± 25	0.045	2.40 ± 1.48–9.23 ± 1.48	4.99 ± 1.13
^232^Th	72 ± 17	0.23	4.37 ± 2.02–32.91 ± 5.54	16.56 ± 3.91
^40^K	1362 ± 83	6.2 × 10^−3^	0.61 ± 0.29–16.06 ± 0.45	8.44 ± 0.52
^137^Cs	43 ± 12	1.3 × 10^−2^	–	0.56 ± 0.16

Table 3 shows committed effective dose values reported for some countries and regions [3, 18–21]. Total adult effective dose from vegetables and fruits frequently produced and consumed in Elazığ Region for ^226^Ra, ^232^Th, ^40^K and ^137^Cs radionuclides were calculated as 20 μSv y^−1^ (SD:3.75) and 30.55 μSv y^−1^ (SD:5.72), respectively. In summary, this study found that adults living in the study region intake a radiation dose of approximately 50.55 μSv y^−1^ from fruit and vegetable consumption. This radiation dose (50.55 μSv y^−1^) is lower than the world average value (290 μSv y^−1^) and presents no risk to public health [3]. Dose values obtained in this present study reflect other reported values in general.

**Table 3:** Average effective dose values for Elazığ Region and its comparison with literature

Region/country	Committed effective dose (μSv y^−1^)			References
	Vegetables	Fruits	Foodstuff	
North America			110	[3]
Asia			110	[3]
Europe			110	[3]
Korean			110	[18]
Jos Plateau/Nigeria			(0.2–2,164)	[19]
Accra metropolitan area/Ghana			4,640	[20]
Rize/Turkey	227	63		[21]
Elazığ/Turkey	20	30.55		Present study

## Conclusion

^226^Ra, ^232^Th, ^40^K and ^137^Cs radionuclide concentrations in vegetables and fruits that are produced and frequently consumed in the Elazığ Region of Turkey were determined in this study. It was found that the radiation dose due to consumption of vegetables and fruits was less than the world average, and poses no threat to public health. The results were lower than the committed effective dose values reported for various regions and countries.
